# Coronary Microvascular Dysfunction in Patients With Systemic Lupus Erythematosus and Chest Pain

**DOI:** 10.3389/fcvm.2022.867155

**Published:** 2022-04-15

**Authors:** Ashley S. Manchanda, Alan C. Kwan, Mariko Ishimori, Louise E. J. Thomson, Debiao Li, Daniel S. Berman, C. Noel Bairey Merz, Caroline Jefferies, Janet Wei

**Affiliations:** ^1^Barbra Streisand Women's Heart Center, Cedars-Sinai Smidt Heart Institute, Cedars-Sinai Medical Center, Los Angeles, CA, United States; ^2^Cedars-Sinai Smidt Heart Institute, Cedars-Sinai Medical Center, Los Angeles, CA, United States; ^3^Biomedical Imaging Research Institute, Cedars-Sinai Medical Center, Los Angeles, CA, United States; ^4^Department of Imaging, Mark Taper Imaging Institute, Cedars-Sinai Medical Center, Los Angeles, CA, United States; ^5^Division of Rheumatology and Department of Biomedical Sciences, Department of Medicine, Cedars-Sinai Medical Center, Los Angeles, CA, United States

**Keywords:** systemic lupus erythematosus, chest pain, coronary microvascular dysfunction, coronary vasospasm, ischemic heart disease

## Abstract

Chest pain is a common symptom in patients with systemic lupus erythematosus, an autoimmune disease that is associated with increased cardiovascular morbidity and mortality. While chest pain mechanisms can be multifactorial and often attributed to non-coronary or non-cardiac cardiac etiologies, emerging evidence suggests that ischemia with no obstructive coronary arteries (INOCA) is a prevalent condition in patients with chest pain and no obstructive coronary artery disease. Coronary microvascular dysfunction is reported in approximately half of SLE patients with suspected INOCA. In this mini review, we highlight the cardiovascular risk assessment, mechanisms of INOCA, and diagnostic approach for patients with SLE and suspected CMD.

## Introduction

Cardiovascular disease (CVD) is a significant contributor to mortality in patients with systemic lupus erythematosus (SLE), with cardiovascular mortality approximately 2.7-fold higher than the general population (1). Despite advancements in treatment for SLE that have improved prognosis, CVD remains a major comorbidity, manifesting as accelerated atherosclerosis and myocardial infarction. Because SLE patients are predominantly female, inadequate CVD risk assessment and underdiagnosis in SLE patients may contribute to CVD morbidity and mortality, which are worse overall for women compared to men with ischemic heart disease (2). Young women with SLE are particularly at risk (3), as those in the 35-to 44-year age group have a 50-fold increase in risk of myocardial infarction compared to age-matched reference women (4). Patients with SLE frequently report chest pain in the absence of obstructive coronary artery disease (CAD), and their chest pain is often attributed to a non-ischemic etiology (5). However, in the past decade, advances in the non-invasive diagnosis of ischemia and no obstructive CAD (INOCA) have determined that coronary microvascular dysfunction (CMD) is highly prevalent in patients with SLE. In this mini review, we highlight the cardiovascular risk assessment, mechanisms of INOCA, and diagnostic approach for patients with SLE and suspected CMD.

## Cardiovascular Risk Assessment in SLE Patients

Traditional cardiovascular risk factors only partially explain the increased CVD risk in SLE patients (6). Thus, traditional cardiovascular risk scores underestimate CVD risk in SLE patients (7). In an analysis of 263 asymptomatic patients with SLE and no prior CVD, risks of non-fatal MI and CVD mortality were over 10-fold higher than what would be expected based on traditional risk factors alone, and presence of SLE increased CVD risk at least 4-fold compared with predictions based on Framingham Risk Score (FRS) models (8). A modified FRS for SLE in which each item is multiplied by 2 was found to be a more accurate predictor of CAD in patients with SLE (9), but this approach does not reflect SLE-specific factors that contribute to heterogeneity of CVD risk in SLE patients. In a cohort of 210 SLE patients without prior CVD or diabetes mellitus (93% female, mean age 45 ± 12 years), both generic risk scores and “SLE-adapted” CVD risk scores underestimated high CVD risk as defined by carotid and femoral atherosclerotic plaque presence (10). Compared to modified risk scores that use a multiplication factor for the presence of SLE (e.g., modified FRS), the QRESEARCH risk estimator version 3 (QRISK3) includes SLE as an independent CVD risk factor and improved prediction of subclinical atherosclerotic CVD (10, 11).

SLE-related factors (e.g., as age at diagnosis, cumulative disease duration, disease activity, and cumulative dose of prednisone or cyclophosphamide) have been found to correlate with coronary atherosclerosis, and elevated c-reactive protein level was associated with CVD events in SLE patients (12, 13). Derivation of a SLE-specific cardiovascular risk equation (SLE-CRE) that incorporates both traditional CVD risk factors and SLE-specific risk factors (SELENA-SLEDAI disease activity score, low C3, and lupus anticoagulant) may better estimate 10-year CVD risk among patients with SLE compared to existing FRS or Pooled Cohort Risk Equation risk scores (14). In a recent single-center analysis of 1,887 SLE patients (88% female, age 39 ± 15 years), SLE-CRE had the highest sensitivity (61%) but lowest specificity (64%) for predicting CVD events compared to QRISK3, FRS, and modified FRS; the authors recommended the modified FRS as the best performer with its simple scoring system (15). However, further studies in larger cohorts are needed to improve the precision of CVD risk assessment in SLE patients. The American College of Cardiology (ACC)/American Heart Association (AHA) prevention guideline recommends to consider the presence of chronic inflammatory or autoimmune disease as a risk-enhancing factor (16) but does not provide specific recommendations regarding risk calculators.

## Mechanisms of Ischemia in Patients with no Obstructive CAD

Chest pain and discomfort are frequently reported by patients with SLE, with high prevalence of angina in comparison to the general population (5). SLE patients with chest pain often present a diagnostic dilemma as they are frequently dismissed when coronary angiography demonstrates non-obstructive CAD or normal epicardial coronary arteries, which is more common among women than men with chest pain (17). Given previous limitations in diagnostic technology, these symptoms are often attributed to psychosomatic pain, chest wall pain, pericarditis/myocarditis, esophageal pain, or myofascial pain (18). Failure to accurately diagnose INOCA has significant clinical implications. Despite the absence of obstructive epicardial CAD, women with persistent stable angina have an elevated risk for CVD events, including all-cause and CVD mortality and progression to obstructive CAD (19, 20). Mechanisms of INOCA are predominantly attributed to coronary microvascular dysfunction (CMD) and/or coronary vasospasm, one or both of which are diagnosed in up to 4 in 5 patients undergoing invasive evaluation for INOCA (21–25). CMD is defined as an attenuated coronary blood flow response or coronary flow reserve (CFR), increased microvascular resistance, microvascular vasospasm, impaired myocardial perfusion reserve, and/or myocardial ischemia in the absence of obstructive CAD (<50% epicardial stenosis or fractional flow reserve >0.80) (26). CMD has an estimated prevalence of approximately 50% in individuals with no obstructive CAD undergoing non-invasive stress tests (27, 28). Epicardial coronary artery spasm is defined as >90% constriction either spontaneously or in response to a provocative stimulus (29).

Endothelial dysfunction, smooth muscle cell dysfunction, and vascular remodeling are major pathogenetic mechanisms in CMD and vasospasm. Endothelial dysfunction results from a reduced production or action of endothelium-derived relaxing factors (nitric oxide, vasodilator prostaglandins, and endothelium-dependent hyperpolarization factors), leading to the inability of the small resistance vessels (prearterioles and arterioles) or the large conduit vessels (epicardial arteries) to vasodilate adequately in response to myocardial demand, therefore leading to ischemia (30). Endothelial dysfunction is caused by aging, hypertension, hyperlipidemia, diabetes, obesity, chronic inflammatory disease, and smoking, and it is a precursor to coronary atherosclerosis (31, 32). In addition, patients with CMD and/or vasospasm may have enhanced coronary vasoconstrictive reactivity related to inflammation, rho kinase-induced myosin light-chain phosphorylation, and increased production of vasoconstrictive mediators (e.g., endothelin-1, serotonin, catecholamines), contributing to smooth muscle cell hyper-reactivity (30). The autonomic nervous system, a key regulator of vascular tone, can also contribute to coronary vasomotor dysfunction, such as heightened sympathetic nervous system activity and increased vagal tone in patients with vasospastic angina, and may be implicated in SLE (33). Finally, vascular remodeling and capillary rarefaction may occur in patients with hypertensive heart disease, aortic stenosis, infiltrative heart diseases, or chronic kidney disease (34). These functional and structural alterations of the coronary microvascular and epicardial arterial system contribute to the imbalance of vasodilating and vasoconstricting responses to stress, resulting in a supply-demand mismatch in coronary blood flow and ischemia.

Microvascular involvement is known to affect multiple organ systems in SLE, for example, in the kidney (lupus nephritis), digestive system (intestinal vasculitis), pulmonary vasculature (pulmonary hypertension, pulmonary vasculitis) and skin (livedo reticularis, cutaneous vasculitis). Although cardiac microvascular involvement occurs in other autoimmune diseases (such as systemic sclerosis and rheumatoid arthritis), the possibility of CMD in SLE has not been well established (35–39). Brachial endothelial dysfunction is significantly impaired in young SLE patients (40). Recent studies have indicated that skin microvascular dysfunction is present in young SLE patients with and without CVD risk factors (41) and associated with elevated carotid intimal media thickness and aortic atherosclerosis (42). Given this propensity for SLE to affect the microvasculature and the endothelium, the cardiovascular involvement is an important consideration beyond the well-known risk for atherosclerotic CAD.

## Inflammatory Mechanisms of SLE Coronary Vascular Dysfunction and Atherosclerosis

**Figure 1:**
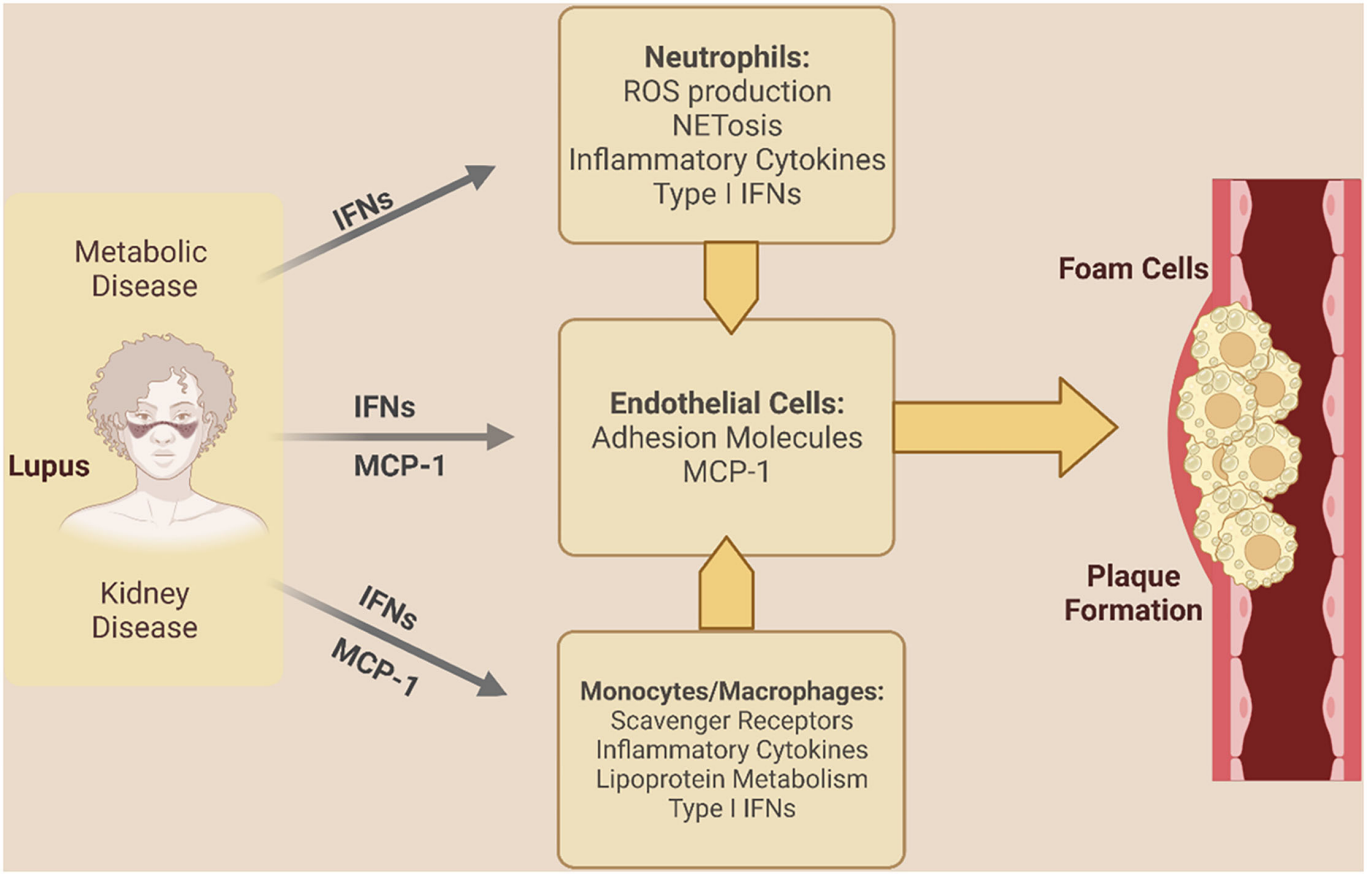
Inflammatory mechanisms driving coronary vascular dysfunction in SLE. Lupus-specific and non-lupus specific factors drive inflammation and coronary vascular dysfunction in SLE. IFNs indicate interferons; MCP-1, monocyte chemoattractant protein 1; NET, neutrophil extracellular trap; ROS, reactive oxygen species. [Created in Biorender. Adapted from (43)].

The chronic inflammatory state of SLE plays a crucial role in accelerating endothelial dysfunction, atherosclerosis, and autonomic dysfunction. Endothelial dysfunction and early atherosclerosis in SLE have been attributed to dysregulation of prothrombotic cell death, and inflammatory mediators (Figure 1) (43–50).

### Monocyte/Macrophage Function

In SLE patients, type I interferons (IFNs) drive an increase in elevated inflammatory chemokines monocyte chemotactic protein-1 [MCP-1] and macrophage inflammatory protein 1α, triggering recruitment of monocytes into the subendothelial space and the upregulation of scavenger receptors such as CD36 (elevated in SLE monocytes) and scavenger receptor A (SR-A) (51–54). IFNs also promote formation of macrophage foam cells with the uptake of oxidized low-density lipoprotein (55–58). Indeed, a correlation between MCP-1 levels and carotid artery intima thickness is observed in SLE, underscoring its potential importance in promoting plaque development in SLE (59).

### Neutrophil Function

Type I IFNs and other inflammatory mediators elevated in SLE also contribute to vascular damage by inducing endothelial dysfunction and reduced nitric oxide mediated vasodilation (60–63). Neutrophils in SLE are a potent source of type I IFNs–either releasing it directly themselves or triggering its production via the release of neutrophil extracellular traps or NETs (64–67). In SLE, NETs from inflammatory neutrophils termed low density neutrophils (LDNs) or granulocytes have been found to contain matrix metalloproteinases, resulting in damage to endothelial cells and drive the release of inflammatory cytokines and type I IFNs, promoting endothelial dysfunction (68–71). Levels of LDNs are associated with coronary plaque burden and endothelial dysfunction (72, 73), thus underscoring the role for these neutrophils in SLE-associated cardiovascular disease and potentially CMD.

### Metabolic Dysregulation

Among SLE patients, renal dysfunction and higher corticosteroid doses are associated with metabolic syndrome, prevalence of which is enhanced in SLE patients and further contributes to increased CVD risk (74). Body mass index is significantly related to insulin resistance in SLE patients, independently of age, sex, race, and corticosteroid use (75). Chronic inflammation may be a potential contributor to enhanced risk of developing metabolic syndrome and insulin resistance in SLE patients, via TNFα release from adipocytes (76–78). In addition to secreting pro-inflammatory cytokines, white adipose tissue also secretes adipokines such as leptin and adiponectin, both of which are responsible for regulating energy homeostasis and metabolism. Leptin also drives inflammation and upregulates oxidative stress, not only in neutrophils and monocytes, but also in endothelial cells and cardiomyocytes (79–82). Indeed, leptin levels are increased in SLE, with evidence that the increase confers enhanced risk of atherosclerosis in these patients (83, 84).

### Antiphospholipid Syndrome

Gene profiling has also revealed specific molecular pathways in the pathogenesis of atherosclerosis and CVD in SLE patients with and without antiphospholipid syndrome (APS). Antiphospholipid antibodies are known to trigger inflammatory cascades with increased expression of cytokines, chemokines, and mediators of endothelial dysfunction, as well as accelerate the influx of oxidized low-density lipoproteins into macrophages, promoting atherosclerosis development (85). From microarray expression profiling in monocytes of patients with SLE with and without APS, IgG-anticardiolipin titers were significantly related to inflammatory, endothelial dysfunction, and oxidative stress markers, as well as were independently predicted both atherosclerosis and thrombosis in SLE patients with APS (85).

## Advanced Cardiac Imaging for Diagnosis of CMD in SLE Patients

Advanced cardiac imaging studies in the past decade have improved understanding of CMD prevalence in SLE patients. Stress transthoracic doppler echocardiography (TTDE), positron emission tomography (PET), and cardiac magnetic resonance imaging (CMR) are recommended by society guidelines for the diagnosis of CMD, with test choice guided by local availability and expertise (86, 87).

### Transthoracic Doppler Echocardiography

TTDE is an established method of CMD evaluation, by measuring CFR in the left anterior descending coronary artery (88, 89). In a study including 21 SLE patients (mean age 60 ± 11 years) and 23 control subjects (mean age 65 ± 10 years) with comparable CVD risk factors, coronary artery calcium scores, and no obstructive CAD, the prevalence of CMD (defined as CFR < 2.5) was higher in the SLE group (67%) compared to the control group (26%), with an odds ratio of 16.7 for CMD in SLE patients after adjusting for age, body mass index, anemia, and hemoglobin level (89). TTDE CFR has also been demonstrated to be reduced in young SLE patients (*n* = 18, mean age 29 ± 6 years) compared to age-, sex- and race-matched healthy controls, supporting the hypothesis that coronary microvascular impairment occurs early in SLE patients (90).

### Stress Cardiac Positron Emission Tomography

Stress cardiac PET utilizes radioactive tracers in patients at rest and with vasodilator stress to quantify absolute myocardial blood flow (MBF) and detect impaired myocardial flow reserve (MFR) suggestive of CMD (91). Cardiac PET MFR < 2.0 has been found to be prognostic in both women and men with INOCA and recommended as a diagnostic threshold for CMD (28). In a recent study of 42 middle-aged SLE patients (mean age 61.2 ± 0.5 years, 97% women) with no obstructive CAD who underwent cardiac PET for evaluation of suspected CAD, MFR < 2.0 consistent with CMD was seen in 57.1% of the patients, and global MFR was significantly reduced compared to matched controls (1.9 ± 0.5 vs. 2.4 ± 0.7, *P* < 0.0001) despite a similar degree of non-obstructive CAD burden and similar myocardial blood flow at rest (92). MFR was reduced in the presence or absence of chronic kidney disease, whether due to lupus nephritis or other causes. MFR was not associated with SLE disease duration nor presence of antiphospholipid antibodies. However, MFR was inversely related to SLE disease activity, consistent with a prior PET study of 13 SLE patients (93). Although prognostic utility of PET MFR has not been reported in SLE patients only, several studies have demonstrated that impaired PET MFR predicts adverse cardiovascular events and all-cause mortality in patients with autoimmune rheumatic diseases including SLE (37, 94, 95).

### Stress Cardiac Magnetic Resonance Imaging

Stress CMR has emerged as a diagnostic and prognostic tool for the evaluation of CMD in patients with no obstructive CAD (96, 97), and may be preferred in SLE patients with concern for myocarditis or pericardial disease, given the standing of CMR as a primary modality of diagnosis for these disease processes (98). CMR measures of coronary blood flow include the semiquantitative myocardial perfusion reserve index (MPRI) <1.84 and quantitative MFR < 2.19, which are sensitive and specific for diagnosing CMD in women and men with angina and no obstructive CAD (99, 100). Furthermore, MPRI ≤ 1.47 independently predicts of major adverse cardiac events in patients with INOCA (101). A study of 20 young women with SLE and angina (mean age 40.6 years) found that visually-identified stress-induced circumferential subendocardial hypoperfusion consistent with global endocardial ischemia was more common in the SLE patients compared with age and sex-matched asymptomatic healthy controls (44 vs. 0%, *P* = 0.014) (102). MPRI trended lower in patients with SLE vs. controls (2.0 ± 0.4 vs. 2.3 ± 0.4, *P* = 0.16), despite absence of obstructive CAD with low burden of coronary atherosclerosis and low-to-moderate SLE disease activity in the SLE patients. MPRI did not correlate with SLE duration or SLE disease activity in this relatively healthy SLE cohort, but presence of SLE was found to be a predictor of subepicardial and subendocardial MPRI (102). In a 5-year follow-up study, a majority of the women reported persistent chest pain but only 25% had a decrease in their MPRI, which occurred in the absence of coronary atherosclerosis progression (103). All individuals with improved CMR findings were concomitantly on aspirin, beta-blocker therapy and cholesterol-lowering agents at follow-up, although clinical trials are needed to understand impact of disease modifying agents and optimal preventive therapy in SLE patients with CMD. Long-term studies to determine prognostic utility of CMR MPRI or MPR in SLE patients are needed.

## Invasive Coronary Function Testing for Diagnosis of CMD and Vasospasm

Invasive testing allows for a more comprehensive delineation of CMD vs. vasospasm in patients with INOCA (17), although prevalence of coronary vasomotor dysfunction in SLE patients has not been reported in invasive coronary function studies. Since SLE patients not uncommonly have Raynaud's phenomenon, a vasospastic disorder of the fingers or toes, coronary vasospasm has been hypothesized to contribute to angina in SLE patients, although confirmatory studies are lacking. Invasive measures of CMD predict mortality and major adverse cardiac events, independent of cardiovascular risk factors (104). Acetylcholine provocation of epicardial vasospasm predicts myocardial infarction and repeated angiography (105). Furthermore, stratified medical therapy based on invasive diagnosis of CMD vs. epicardial vasospasm improved angina and quality of life in a randomized clinical trial (21). However, the diagnostic and prognostic utility of invasive coronary function testing for SLE patients is unknown.

## Discussion

**Figure 2:**
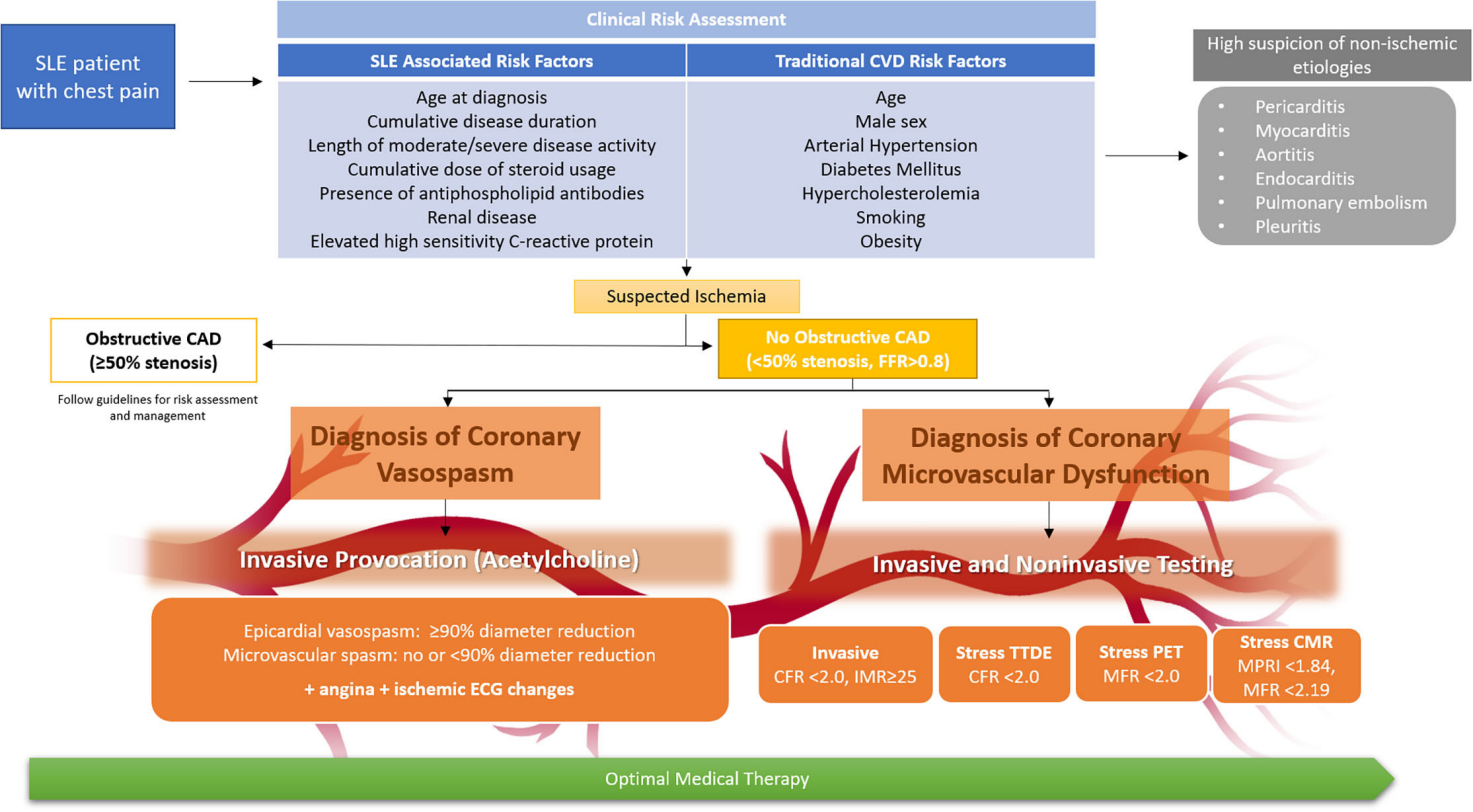
Clinical risk assessment of SLE patients with chest pain and algorithm for diagnosis of coronary microvascular dysfunction and vasospasm. Non-invasive and invasive testing allow assessment of coronary microvascular dysfunction, while invasive acetylcholine provocation testing can additionally assess coronary vasospasm. While CMR may be preferred for concomitant assessment of myocarditis, test choice should be guided by local expertise and availability. All SLE patients with angina should receive optimal medical therapy, including lifestyle intervention and intensive pharmacologic therapy (anti-ischemic and preventive therapy). CAD indicates coronary artery disease; CFR, coronary flow reserve; CMR, cardiac magnetic resonance imaging; CVD, cardiovascular disease; ECG, electrocardiogram; FFR, fractional flow reserve; IMR, index of microcirculatory restriction; MFR, myocardial flow reserve; MPRI, myocardial perfusion reserve index; PET, positron emission tomography; and TTDE, transthoracic doppler echocardiography.

Chest pain is a frequent complaint of SLE patients in the absence of obstructive CAD, and ischemic mechanisms such as CMD and coronary vasospasm should be considered in the diagnostic algorithm. Given the higher risk of CVD mortality and morbidity attributed to SLE due to the inflammatory and metabolic pathophysiologic mechanisms, early identification and prevention of CVD risk factors is warranted, although available risk scores underestimate CVD risk in SLE patients. SLE patients presenting with chest pain should undergo clinical risk assessment and subsequent evaluation for CAD, with the consideration of CMD and/or coronary vasospasm evaluation in those with no obstructive CAD (Figure 2). Society guidelines recommend intensification of preventive and anti-ischemic therapies in patients with stable angina and suspected INOCA, particularly those with known coronary atherosclerosis, but evidence-based treatment specific to INOCA is lacking (86, 87).

CMD evaluation of SLE patients to date consistently demonstrates impaired flow or perfusion reserve compared to matched controls (Table 1). A relationship between CMD and SLE disease activity was reported in several of the studies, but overall no relationship between CMD and disease duration, risk factors, or steroid use, although larger cohorts may be needed to further evaluate predictors of CMD in SLE patients. Blood biomarkers of inflammation were not reported in the majority of studies. Several studies also reported higher resting velocities or myocardial flow in SLE patients compared to controls, suggesting underlying differences in coronary blood flow autoregulation, suspected due to vasomotor and autonomic dysfunction. Although studies comparing CMD in SLE vs. other autoimmune rheumatic diseases are limited, CMD has been reported in autoimmune rheumatic diseases such as rheumatoid arthritis and systemic sclerosis, strengthening the link between inflammation and CMD (36). While the pathogenic inflammatory mechanisms of various autoimmune rheumatic diseases are increasingly described, differences in the inflammatory mechanisms that contribute to CMD are not well understood.

**Table 1:** Review of studies that assess CMD in patients with SLE.

**Imaging modality**	**Reference**	***N***	**Mean age (years)**	**% Female**	**Disease duration (years)**	**Disease activity and prevalence of steroid use**	**Measures of CMD**	**Resting coronary velocity (cm/s) or Myocardial flow (mL/min/g)**	**Other measures of CAD**
TTDE	Kakuta et al. (89)	21 (SLE) 23 (C)	60 ± 11 (SLE) 65 ± 10 (C)	81% (SLE); 78% (C)	9 (3–13)	SLEDAI 0 (0) prednisolone 95.2%	CFR 2.23 ± 0.71 (SLE) CFR 3.01 ± 0.72 (C) CFR not related to age, disease duration, steroid use, hematocrit, CRP	DFV 19.8 ± 5.5 (SLE) DFV 17.1 ± 4.7 (C)	CACS of LAD 0 (0–138) (SLE); CACS of LAD 30 (0–225) (C); total CACS similar between groups; CACS not related to CFR
	Hirata et al. (90)	18 (SLE) 19 (C)	29 ± 6 (SLE) 28 ± 4 (C)	100% (SLE) 100% (C)	8.2 ± 7.2	SLEDAI 11 ± 5	CFR 3.4 ± 0.8 (SLE) CFR 4.5 ± 0.5 (C) CFR not related to SLEDAI, disease duration, CRP, cholesterol, steroid use	DFV 33.6 ± 9.5 (SLE) DFV 26.2± 6.5 (C)	NR
PET	Weber et al. (92)	42 (SLE) 69 (C)	61 ± 0.5 (SLE) 62 ± 12 (C)	97% (SLE) 95% (C)	15.7 ± 10.5	SLEDAI 4 (0–6) prednisone 48%	MFR 1.91 ± 0.5 (SLE) MFR 2.4 ± 0.7 (C) MFR inversely related to SLEDAI but not to disease duration	NR	CAC = 0 in ~50% of each group (MFR remained lower in SLE vs. C); Frequency of CAC severity similar between groups
	Recio-Mayoral et al. (93)	13 (SLE) 12 (RA) 25 (C)	30 ± 8 (SLE) 47 ± 7 (RA) 44 ± 9 (C)	100% (SLE) 83% (RA) 80% (C)	11 ± 7 (SLE) 16 ± 11 (RA)	SLEDAI 0 (0–2) DAS-28 2.0 (1.7–2.5) prednisone 42% (SLE), 61% (RA)	MFR 2.44 ± 0.78 (SLE + RA) MFR 3.87 ± 0.92 (C) Similar MFR between SLE and RA; MFR inversely related to SLEDAI and disease duration (SLE+RA) but not to age, prednisone dose	MBF 1.25 ± 0.27 (SLE + RA) MBF 1.13 ± 0.27 (C)	normal coronaries (72%) mild CAD (28%) obstructive CAD (0%)
	Weber et al. (94)	41 (SLE) 63 (psoriasis) 94 (RA)	65 ± 12 (all)	80% (all)	NR	NR	MFR 1.83 (1.6–2.2) (SLE) MFR 1.80 (1.4–2.5) (psoriasis) MFR 1.93 (1.5–2.2) (RA) MFR similar between groups	MBF 1.01 (0.88–1.40) (SLE) MBF 0.99 (0.8–1.3) (psoriasis) MBF 1.03 (0.82–1.3) (RA) MBF similar between groups	NR
	Feher et al. (95)	101 (ARD) 101 (C)	63 (56–69) (ARD) 60 (52–70) (C)	80% (ARD) 87% (C)	NR	NR	MFR 1.68 (1.34–2.05) (ARD) MFR 1.86 (1.58–2.28) (C)	MBF 1.00 (0.84–1.21) (ARD) MBF 0.80 (0.68-0.88) (C)	CAC>0 (50%) (ARD) CAC>0 (39%) (C)
CMR	Ishimori et al. (102)	20 (SLE) 10 (C)	41 ± 11 (SLE) 53 ± 5 (C)	100%	12.8	SLEDAI 0 (*n* = 3), 1–5 (*n* = 10), 6–10 (*n* = 5) corticosteroid within 1 year 80%	MPRI 2.0 ± 0.4 (SLE) MPRI 2.3 ± 0.4 (C) MPRI not related to SLEDAI or SLE duration	NR	normal coronaries (89%) mild CAD (11%) obstructive CAD (0%)
	Sandhu et al. (103)	20 (SLE)	41 (baseline) 46 (follow-up)	same as above	baseline: same as above	baseline: same as above follow-up: SLEDAI 0 (*n* = 5), 1–5 (*n* = 8), 6–10 (*n* = 3), >10 (*n* = 1) corticosteroid within 1 year 41%	MPRI 2.0 ± 0.4 (baseline) MPRI 2.1 ± 0.6 (follow-up) MPRI similar at baseline and follow-up (36% with persistent CMD)	NR	progression to mild or obstructive CAD (7%) no change (93%)

*Data are expressed as mean ± SD, or as median (IQR), or percentages as specified. NR indicates data not reported in the study. ARD indicates autoimmune rheumatic disease; C, controls; CAC, coronary artery calcium; CAD, coronary artery disease; CFR, coronary flow reserve; CMR, cardiac magnetic resonance imaging; DAS-28, Disease Activity Score for rheumatoid arthritis; DFV, diastolic flow velocity; LAD, left anterior descending artery; MBF, myocardial blood flow; MFR, myocardial flow reserve; MPRI, myocardial perfusion reserve index; PET, positron emission tomography; SLE, systemic lupus erythematosus; SLEDAI, Systemic Lupus Erythematosus Disease Activity Index; and TTDE, transthoracic doppler echocardiography*.

Significant knowledge gaps exist in SLE patients with INOCA, including the prevalence of coronary vasospasm, contribution of autonomic dysfunction to ischemia and chest pain, role of disease-modifying antirheumatic drugs on CMD and cardiovascular outcomes, non-invasive and invasive strategies to identify high risk patients for targeted preventive therapy, and optimal therapy of microvascular and vasospastic angina. While inflammatory mechanisms of SLE are increasingly understood, mechanistic pathways underlying the pathobiology of SLE-specific coronary vasomotor dysfunction remain unknown. Future prospective research studies are needed to address these questions in the risk assessment. diagnosis and treatment of SLE patients with INOCA.

## Author Contributions

All authors contributed with conceptualization, writing—review and editing, validation, and funding. All authors contributed to the article and approved the submitted version.

## Funding

This work was supported by research funding from NIH R01HL146158 (CB and JW), R01HL124649 (CB), R01HL153500 (JW), U54AG065141 (CB), K23HL125941 (JW), R01AI164504 (CJ), the Office of the Assistant Secretary of Defense for Health Affairs through the Department of Defense Lupus Research Program Award Number W81XWH-18-1-0709 (CJ), the Barbra Streisand Women's Cardiovascular Research and Education Program (CB), the Linda Joy Pollin Women's Heart Health Program (CB), the Erika Glazer Women's Heart Health Project (CB), and the Adelson Family Foundation (CB).

## Conflict of Interest

CB has served as consultant for Sanofi, Abbott Diagnostics, and iRhythm. DB has served as a consultant for Bayer and received software royalties from Cedars-Sinai Medical Center. JW has served as consultant and advisory board member for Abbott Vascular. The remaining authors declare that the research was conducted in the absence of any commercial or financial relationships that could be construed as a potential conflict of interest.

## Publisher's Note

All claims expressed in this article are solely those of the authors and do not necessarily represent those of their affiliated organizations, or those of the publisher, the editors and the reviewers. Any product that may be evaluated in this article, or claim that may be made by its manufacturer, is not guaranteed or endorsed by the publisher.
